# Toward Real-Time Automated Detection of Turns during Gait Using Wearable Inertial Measurement Units

**DOI:** 10.3390/s141018800

**Published:** 2014-10-10

**Authors:** Domen Novak, Maja Goršič, Janez Podobnik, Marko Munih

**Affiliations:** 1 Sensory-Motor Systems Lab, ETH Zurich, Tannenstrasse 1, CH-8092 Zurich, Switzerland; 2 Laboratory of Robotics, University of Ljubljana, Tržaška cesta 25, SI-1000 Ljubljana, Slovenia; E-Mails: maja.gorsic@robo.fe.uni-lj.si (M.G.); janez.podobnik@robo.fe.uni-lj.si (J.P.); marko@robo.fe.uni-lj.si (M.M).

**Keywords:** gait analysis, inertial measurement units, gait event detection, wearable sensors, wireless sensor networks

## Abstract

Previous studies have presented algorithms for detection of turns during gait using wearable sensors, but those algorithms were not built for real-time use. This paper therefore investigates the optimal approach for real-time detection of planned turns during gait using wearable inertial measurement units. Several different sensor positions (head, back and legs) and three different detection criteria (orientation, angular velocity and both) are compared with regard to their ability to correctly detect turn onset. Furthermore, the different sensor positions are compared with regard to their ability to predict the turn direction and amplitude. The evaluation was performed on ten healthy subjects who performed left/right turns at three amplitudes (22, 45 and 90 degrees). Results showed that turn onset can be most accurately detected with sensors on the back and using a combination of orientation and angular velocity. The same setup also gives the best prediction of turn direction and amplitude. Preliminary measurements with a single amputee were also performed and highlighted important differences such as slower turning that need to be taken into account.

## 1. Introduction

State-of-the-art powered lower limb exoskeletons and prostheses are able to switch between different operating modes depending on the user’s intended activities. For example, a powered prosthesis can provide different types of support depending on whether the user wishes to walk, stand in place or sit down [1]. While this switching can be done using sensors built into the assistive device itself, many studies have examined the possibility of using additional wearable sensors such as inertial measurement units (IMUs – a combination of accelerometer, gyroscope and, optionally, a magnetometer) or pressure-sensitive insoles to detect intended actions.

Most gait analysis studies with wearable sensors have focused on steady-state walking, where they extract parameters such as stride length [2] and detect transitions between different gait phases such as toe-off and heel-strike [3–9]. A smaller number of studies has also focused on detecting transitions between walking and standing or sitting [10–12]. When used with an assistive device, transitions between gait phases and transitions to/from walking should be detected as soon as possible so that the assistive device has enough time to apply smooth and effective support to the user [5,13]. Aside from supporting gait, the device can also enhance gait stability by detecting and counteracting potential stumbling [14].

Most automated gait analysis algorithms treat gait as taking place only in the sagittal plane of the body, allowing considerably simplified calculations [11,12]. However, this could lead to problems if the user wishes to turn while walking with an assistive device. The device should also be able to detect turns, which would require measurements outside the sagittal plane. As with detection of gait phase transitions, the detection should be as fast as possible so that the assistive device can provide support. Such rapid turn detection algorithms have been emphasized as a potentially important component of future assistive orthoses and prostheses [15].

Numerous personal navigation algorithms have been developed for wearable sensors to track and reconstruct walking trajectories over longer distances [16,17]. While such algorithms can also be used during turns, they do not provide automated turn detection, and they are not optimized for the early turn detection that would be needed for an assistive device to react rapidly. An early algorithm for automated detection of turns using stationary cameras was demonstrated by Olivier and Cretual [18], but the algorithm was not well-evaluated and is not transferrable to wearable sensors.

Two approaches for automated turn detection using wearable sensors were recently presented: by Mariani *et al.* [19] for a foot-worn IMU and by El-Gohary *et al.* [20] for a pelvis-worn IMU. In the approach of Mariani *et al.* [19], the difference in IMU orientation between two consecutive steps is computed at each step, and a turn is considered as detected if this difference is greater than 20 degrees around the vertical axis. In the approach of El-Gohary *et al.* [20], a turn can last several steps and is considered detected when the measured angular velocity around the vertical axis exceeds 15°/s, but post-hoc analysis must be done to verify that a turn has actually occurred. Both of these studies are optimized for offline turn annotation and therefore have similar weaknesses:

- Using an orientation threshold [19] means that a significant part of the turn must have already occurred before it is detected. In principle, a turn could be detected earlier by detecting the onset of high angular velocity, as suggested by El-Gohary *et al.* [20]. Early detection would also mean more time for an assistive device to react and support the turn. However, as an angular velocity threshold is prone to more false positives, El-Gohary *et al.* [20] include an additional orientation threshold in postprocessing. All thresholds are selected manually for the purpose of offline annotation, so it is unclear what approach is more suitable for real-time turn detection.
- Only a single IMU placement (foot or pelvis) is tested. While this does not matter for offline turn detection, healthy adults of various ages generally employ a “top-down” turning strategy while walking (*i.e*., not turning on the spot): starting with reorientation of the head followed by the trunk, pelvis and finally feet [21,22]. Although some pathologies such as Parkinson’s disease exhibit simultaneous head, trunk and pelvis reorientation [22], this nonetheless suggests that IMUs placed on the head or trunk may allow faster detection of turns during gait and would be preferable to IMUs on the pelvis or foot.

Our work attempts to improve existing turn detection algorithms in order to make them more suitable for real-time use with an assistive device. Specifically, we wish to answer two questions:

- Where should sensors be placed on the body to provide the most rapid and reliable turn detection?
- Can rapid turn detection be best performed with an orientation threshold, an angular velocity threshold, or a combination of the two?

To answer these questions, we primarily performed an evaluation with unimpaired subjects, though a single pilot test was done with an amputee. In a final application, the algorithm would detect turns in real time and send this information to an assistive device (prosthesis or exoskeleton) that would intelligently support the turn.

## 2. Experimental Section

### 2.1. Subjects

As we have very limited access to amputee subjects, the majority of the study was performed with subjects who had no physical or cognitive abnormalities that would affect gait. Ten such subjects participated (9 males, 1 female, mean age 30.9 years, standard deviation 4.3 years), with measurements carried out at the Laboratory of Robotics, University of Ljubljana, Slovenia. Sections 2.2–2.5 describe the measurements with the unimpaired subjects.

Following tests with unimpaired subjects, a pilot test was performed with a male transfemoral amputee whose right limb had been amputated in 2003. He was 66 years old, 180 cm tall and wore a C-Leg prosthesis (Otto Bock, Germany) with an Icelandic-Swedish-New York above-knee socket. This measurement was carried out at the Fondazione Don Gnocchi in Florence, Italy. Due to logistical reasons, it was much more limited than tests with unimpaired subjects and is described in Section 2.6.

The same experimenters carried out tests with both healthy subjects and the amputee. The measurements were approved by relevant ethics committees in Slovenia and Italy. All subjects signed an informed consent form after the purpose and procedure of the experiment had been explained to them.

### 2.2. Measurement Protocol

**Figure 1.**
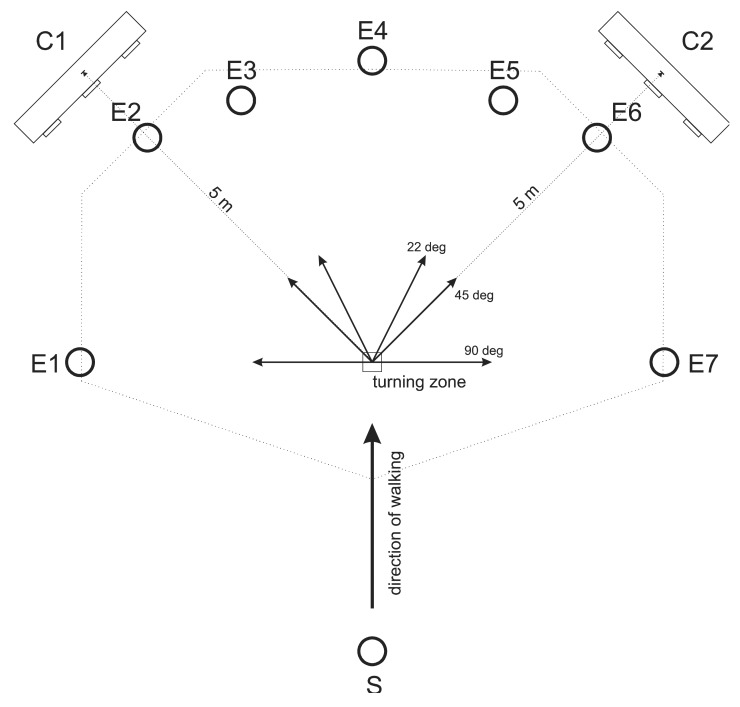
Top view of the experimental set-up. Subjects started at point S and walked straight ahead for about 5 m until they reached the turning zone (square), at which point they followed one of the seven possible paths until they reached the end points (E1–E7). C1 and C2 represent cameras used as the optical reference system.

The measurement protocol was inspired by the protocol of Akram *et al.* [21]. Each subject performed 49 trials at his/her natural walking speed. A mark on the lab floor indicated the “turning zone”. In each trial, subjects first stood still for approximately 5 s, then received a verbal signal to begin walking straight from one end of the lab toward the turning zone. Upon reaching the approximate turning zone, there were seven possibilities: they could continue walking straight, turn left by 22, 45 or 90 degrees, or turn right by 22, 45 or 90 degrees. 45 and 90 degrees represent commonly studied turning angles while 22 degrees was added as a greater challenge for the turn detection algorithm. Visible markings placed at eye-height at the end of the potential travel paths defined the direction of each turn. Upon reaching the end of the travel path, the subject returned to the starting point and began the next trial. The total 49 trials were divided into 7 consecutive blocks of 7 trials, with each block containing all 7 possible travel paths presented in random order. Before beginning each trial, the subject was told the travel path (turn direction) for the next trial. Subjects were also instructed to look at the ground as little as possible. Figure 1 shows the experimental set-up with the seven possible travel paths.

### 2.3. Sensors

#### 2.3.1. Inertial Measurement Units

**Figure 2.**
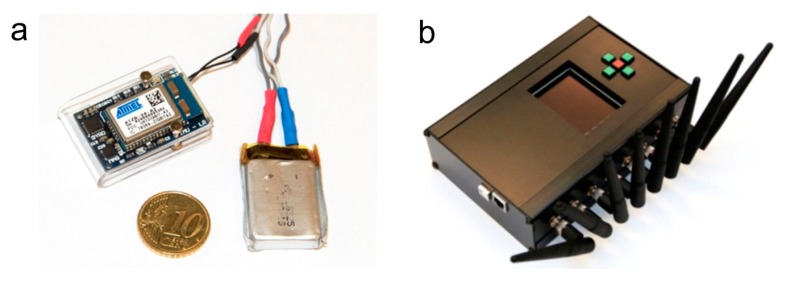
A single inertial measurement unit with battery (left) and the central wireless receiver (right).

The IMUs were developed in our laboratory using commercial components. Each one contains an Invensense triaxial gyroscope (measures angular velocity), STMicroelectronics triaxial accelerometer (measures linear acceleration) and Honeywell triaxial magnetometer (measures direction of Earth’s magnetic field). In addition to the sensors, the IMU also contains a Wellpower battery and Atmel ZigBit wireless transceiver. Each IMU transmits raw accelerometer, gyroscope and magnetometer outputs wirelessly to a central unit at a frequency of 100 Hz and a worst-case transmission delay of 20 ms [11]. These raw outputs can also be fused into an IMU orientation estimate using a Kalman filter [23], with a maximum expected orientation error of 3 degrees [11]. The working range of the wireless transmission is approximately 15 m. Further technical details are available in our previous papers [11,23]. A photo of an IMU and the central unit is shown in Figure 2. This IMU design is comparable to commercial sensors by companies such as Xsens Technologies (Netherlands) or INSENCO (Japan) as well as research sensors such as the ReSense [24], and should not be considered novel on its own.

**Figure 3.**
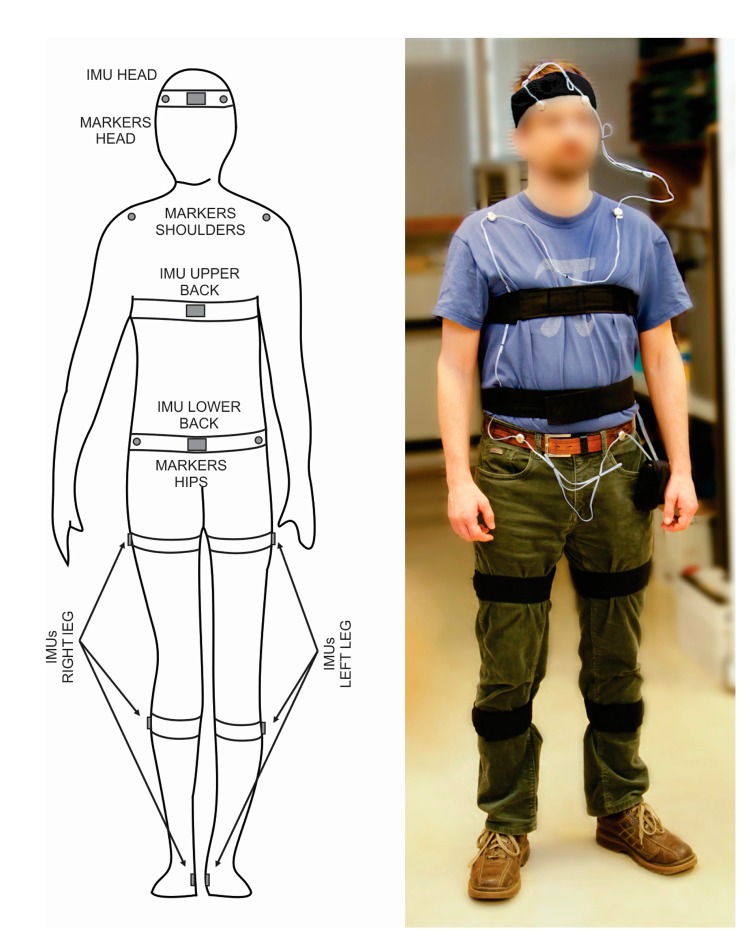
IMU and Optotrak marker placement on the body: schematic (**left**) and photograph (**right**).

Nine IMUs were placed on the body in total. Six were placed on the legs (on each foot, shank and thigh), one was placed on the lower back, one was placed on the upper back, and one was placed on the head. The IMU positions are shown in Figure 3. IMU placement on each segment was performed based on the original recommendations for these IMUs [23] while the transformation from the IMU’s to the body segment’s coordinate system was done based on updated recommendations of Šlajpah *et al.* [25]. Following recommendations from previous studies [26,27], any movable ferromagnetic materials were moved at least 1 m from the experiment area, and measurements of the magnetic field variation inside the lab were performed prior to the study. No significant magnetic field changes were found in the horizontal plane over the experimental area, though some disturbances did occur close to the ground and may affect the output of the magnetometer on the foot. These disturbances were not controlled, as they would also occur in real-world conditions.

In principle, each IMU has 12 output signals: accelerometer, gyroscope and magnetometer signals for three axes as well as orientation for three axes. However, as we only wish to detect left/right turns, 3 signals are used for each IMU: angular velocity (raw gyroscope output), angle (Kalman filter output) and magnetometer output around the axis that indicates left/right movement. For each trial, the angle and magnetometer values at the start of the trial (before the subject begins walking) were subtracted from the values recorded during the trial, effectively setting the initial value to zero.

#### 2.3.2. Optical Reference System

To obtain the reference time and direction of each turn, two Optotrak Certus cameras (Northern Digital Inc., Waterloo, Canada) were placed in the room as shown in Figure 1. This positioning was chosen to cover the maximum possible area around the turning zone. Six infrared markers were placed on the subject as shown in Figure 3 in order to measure head, shoulder and hip orientations at 100 Hz. While two markers per body segment are not sufficient to capture three-dimensional kinematics, they are sufficient to detect the turn onset [21,28].

For each trial, two human experts were independently asked to manually determine the turn onset by examining the Optotrak-recorded head, shoulder and hip orientations simultaneously. Turn onset was defined as the moment when the turn actually begins occurring. If the difference between the two manually defined onsets was less than 200 ms, the mean of the two onsets was used as the reference turn onset for that trial. If the difference was more than 200 ms, the two experts were asked to re-examine the trial together and reach a consensus. This occurred in approximately 5% of all trials.

### 2.4. Turn Detection Algorithm

The turn detection algorithm can in principle be used with any of the 9 IMUs. It consists of three components: determining the turn onset (moment when the turn begins occurring), determining the direction of the turn, and determining the approximate amplitude of the turn. The turn onset is detected by optimizing parameters of manually-defined rules while the direction and amplitude are determined using supervised machine learning.

#### 2.4.1. Turn Onset

The reasoning behind the turn onset detection algorithm is as follows: when the subject begins turning, the body segments reorient themselves to the new gait direction, which should be measurable by measuring angular acceleration, velocity or displacement around the vertical axis. Using an IMU, which directly measures angular velocity and uses a Kalman filter to calculate orientation around the vertical axis, we have three possibilities:

- A turn is considered detected when the orientation around the vertical axis has changed by a sufficient value, as done by Mariani *et al.* [19]. However, this requires a large part of the turn to have already occurred, leaving less time for an assistive device to react.
- A turn is considered detected when a sufficiently high angular velocity is measured by the gyroscope, as done by El-Gohary *et al.* [20]. This high velocity should be visible in the gyroscope output before the walking direction has changed significantly. Furthermore, such a turn detection algorithm would only require the gyroscope, so the accelerometer and magnetometer could be omitted. However, if the turn is small or slow enough, the angular velocity may not be large enough to allow reliable turn detection.
- A turn is considered detected when a sufficiently high angular velocity is measured by the gyroscope or the orientation around the vertical axis has changed by a sufficient value. This is essentially a combination of the above two approaches and may yield better performance for small or slow turns: if the turn is not detected from the angular velocity, it is still detected slightly later when the orientation has sufficiently changed.

The third type of turn onset detection algorithm can be described in pseudocode as:
while *turn_has_occurred* = 0{ if abs(*angular_velocity*) > *gyr_thresh turn_has_occurred* = 1; else  if abs(*current_angle*) > *angle_thresh turn_has_occurred* = 1;  end end}

The turn onset is the moment when *turn_has_occurred* changes from 0 to 1. To create an algorithm based only on angular velocity or only on orientation, we simply remove one of the two threshold checks (on *gyr_thresh* or *angle_thresh*).

To use the turn onset detection algorithm, we must only define the thresholds *gyr_thresh* and *angle_thresh*. This can be easily done with a training database of trials with known turn onsets. The optimal *gyr_thresh* and *angle_thresh* are then those that minimize the difference between reference and detected turn onsets.

We constructed a cost function that needs to be minimized in order to obtain the *gyr_thresh* and *angle_thresh* thresholds. Our assumptions for this cost function were:

- As manually-defined turn onsets may be earlier than is detectable by an IMU, no penalty is given if the difference between reference and detected turn onset is smaller than 500 ms. This 500-ms value was selected arbitrarily, and larger or smaller values could be chosen depending on the desired strictness.
- Premature detections (more than 500 ms before reference), late detections (more than 500 ms after reference) and false negatives (turn not detected) are penalized equally—we do not care more about a particular error type. We also do not care how premature or late the detection is; a turn detected 501 ms before the reference is penalized equally to one detected 5000 ms before the reference.
- We also wish to avoid false positives. For this reason, the training database contains examples of the subject walking straight without turning. If a turn is detected for such a trial, it receives the same penalty as a premature or late detection.

The cost function can be described in pseudocode:
function *num_errors* = costfunction(*gyr_thresh, angle_thresh*){ *num_errors* = 0; for all trials in database  *detected_time* = turn_detection_algorithm(*IMU_signals, gyr_thresh, angle_thresh*);  if trial contains turn   if turn was detected    if abs(*detected_time* – *reference_time*) > 500 ms *num_errors* = *num_errors* + 1;    end   else    *num_errors* = *num_errors* + 1;   end  else   if turn was detected    *num_errors* = *num_errors* + 1;   end  end end}

We optimized this cost function using a genetic algorithm (implemented via MATLAB 2012a’s Optimization Toolbox). In principle, the different errors could also be weighted differently depending on the application; for example, if the goal is to minimize the amount of false negatives, we could add a higher number to *num_errors* for each false negative.

#### 2.4.2. Turn Direction and Amplitude

Once a turn has been detected, the algorithm classifies the turn direction (left/right). This is done using a classifier with three inputs:

- Angular velocity around the vertical axis at time *T*,
- Magnetometer output around the vertical axis at time *T*,
- Orientation around the vertical axis at time *T*.

The classifier’s output is then the turn direction. *T* can be the detected turn onset time (*T_ONSET_*), but can also be a later time (e.g., *T_ONSET_* + 100 ms), and different values of *T* were tested in crossvalidation (Section 2.5.2). For the classifier, we used linear discriminant analysis (implemented via MATLAB 2012a’s *classify* function). It was trained with a database of trials with known turn directions. For each training trial, the reference inputs are the above three inputs obtained at the same time with respect to turn onset in that trial (e.g., at *T* = *T_ONSET_* + 100 ms, with *T_ONSET_* being different for each trial).

Similarly, the turn amplitude (22/45/90 degrees) can be classified using a classifier with three inputs:

- Absolute angular velocity around the vertical axis at time *T*,
- Absolute magnetometer output around the vertical axis at time *T*,
- Absolute orientation around the vertical axis at time *T*.

In this case, we use the absolute values of the signals: positive values indicate turning in one direction while negative values indicate turning in the other, but we only care about the amplitude. For the classifier, we again used linear discriminant analysis and the same training database.

### 2.5. Performance Evaluation

#### 2.5.1. Turn Onset Detection

As data were recorded in advance, the algorithm was evaluated offline using within-subject and subject-independent leave-one-out crossvalidation. In subject-independent crossvalidation, recorded trials from all but one subject are used to train the algorithm (set the thresholds and train the classifiers), which is then tested on the remaining subject. The procedure is repeated as many times as there are subjects, and results are averaged across all subjects. Within-subject crossvalidation, on the other hand, is done for each subject separately. All but one trial from the subject are used to train the algorithm, which is then tested on the remaining trial. This is repeated as many times as there are trials for that subject. The entire procedure is performed for each individual subject, and results are averaged across all subjects. Since each subject performed 49 trials and there were 10 subjects in total, the training database consisted of 48 trials in within-subject crossvalidation and 441 (49 times 9) trials in subject-independent crossvalidation.

The goal of the study was to compare different IMU positions as well as different criteria for turn onset detection. We therefore trained and evaluated the turn onset detection separately for six IMUs: the head, the upper back, the lower back, the left thigh, the left shank and the left foot (IMUs on the right leg gave similar results). We also trained and evaluated it separately for the three possible criteria: *gyr_thresh only, angle_thresh* only, and both *gyr_thresh* and *angle_thresh*. In total, 6 (IMU position) × 3 (onset detection criterion) × 2 (within-subject or subject-independent crossvalidation) separate evaluations were performed.

As the evaluation outcome, we calculated the:

- *Percentage of correct detections* (CD) – number of trials where a turn is detected within 500 ms of the reference, divided by number of trials where a turn occurs.
- *Percentage of premature and late detections* (PLD) – number of trials where a turn is detected more than 500 ms earlier or later than the reference, divided by number of trials where a turn occurs.
- *Percentage of false negatives* (FN) – number of trials where a turn occurs but is not detected, divided by number of trials where a turn occurs. The sum of CD, PLD and FN is always 100%.
- *Percentage of false positives* (FP) – number of trials where a turn is detected but does not actually occur, divided by number of trials where a turn does not occur.
- *Mean difference between detected and reference onset* (DIFF) in milliseconds, with a positive number meaning that onset was on average detected later than reference onset. Calculated over trials with either a correct, premature or late detection.

For the case of onset detection using both gyroscope and orientation thresholds, we furthermore tested a special case: onset detection when 22-degree turns are removed from the database and, as an extreme case, when both 22 and 45-degree turns are removed. As smaller turns are more difficult to detect than large ones, this gives the results for potential applications where we would not care about small turns and would only wish to detect larger ones.

#### 2.5.2. Turn Direction and Amplitude

Direction and amplitude classification were evaluated as:

- *Percentage of correctly classified directions at T* = *T_ONSET_* + [0, 0.1, 0.2, 0.3, 0.4, 0.5] s,
- *Percentage of correctly classified amplitudes at T* = *T_ONSET_* + [0, 0.1, 0.2, 0.3, 0.4, 0.5] s.

These were calculated at different times since we assume that the turn direction and amplitude are easier to identify later in the turn. They were calculated only for trials where a turn is detected correctly (within 500 ms of the reference).

### 2.6. Pilot Test with Amputee

Since we lack access to amputee subjects, the pilot test with the amputee was performed at a different location by the same experimenters on the same day as a larger set of measurements for an unrelated gait analysis study. The amputee wore IMUs on the lower back, thighs, shanks and feet like the unimpaired subjects, but upper back and head IMUs were omitted for logistical reasons. Furthermore, due to time constraints, only 12 trials were performed: two for each turn direction (left/right) and amplitude (22/45/90 degrees).

Since there were not enough trials to perform within-subject crossvalidation with the amputee, only a form of subject-independent crossvalidation was performed: the onset detection and direction/amplitude classification algorithms were trained using data from all healthy subjects and tested on the 12 amputee trials. Results are given in Section 3.3.

## 3. Results

**Figure 4.**
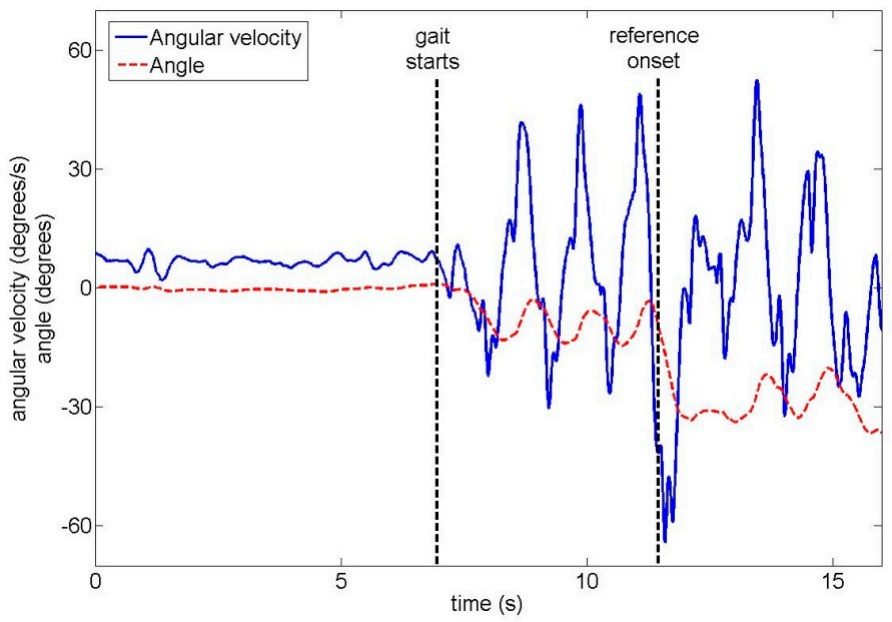
An unimpaired subject performing a 22-degree left turn, with two signals from the upper back IMU: the angular velocity and the angle around the vertical axis.

**Figure 5.**
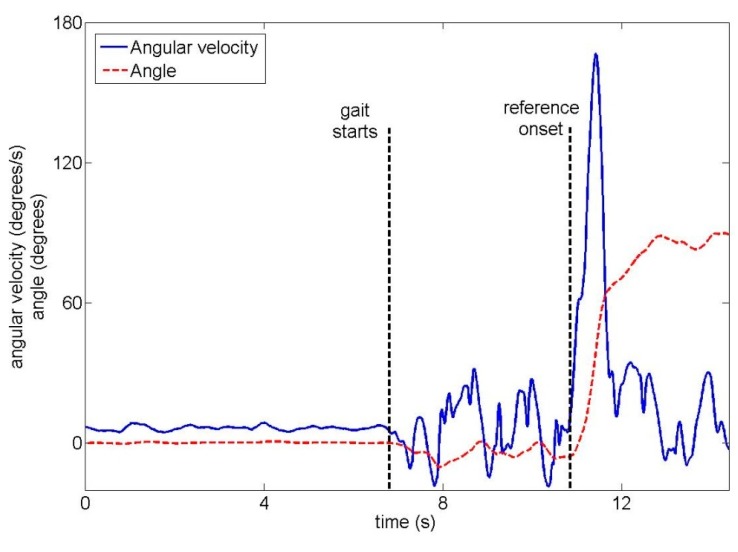
An unimpaired subject performing a 90-degree right turn, with two signals from the upper back IMU: the angular velocity and the angle around the vertical axis.

The total duration of one trial across all ten unimpaired subjects was 16.4 s ± 2.2 s (mean ± standard deviation). Subjects began walking 8.3 s ± 2.3 s after the start of the trial. In trials where a turn occurred, turn onset was 10.2 s ± 2.3 s after the start of the trial. Examples of the upper back gyroscope signal and angle are shown for unimpaired subjects in Figure 4 (22-degree left turn) and Figure 5 (90-degree right turn).

### 3.1. Unimpaired Subjects: Turn Onset

In this section, we present onset detection results for the three different criteria: orientation threshold only (Table 1), angular velocity threshold only (Table 2) and combination of both thresholds (Table 3). Each table contains results for both crossvalidation types and for all IMU positions.

**Figure 6.**
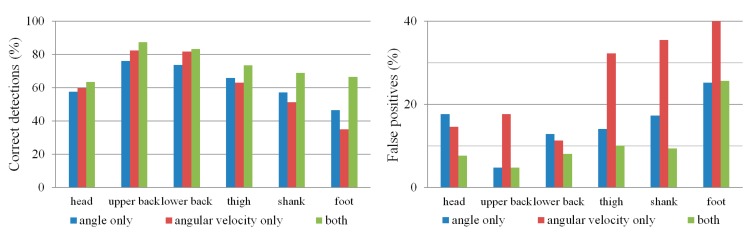
Percentage of correct detections (left) and false positives (right) for different IMUs and detection criteria. Results are shown for within-subject crossvalidation only.

Comparing the results in Tables 1 and 2 shows that the angular velocity threshold has a slightly higher percentage of correct detections and can detect turn onset earlier than the orientation threshold, but at the cost of more false negatives and false positives. However, a combination of both thresholds (Table 3) provides the highest percentage of correct detections together with a low percentage of false negatives and false positives. With regard to IMU position, the upper and lower back IMUs give the best results, with lower accuracy for the IMUs on the legs and the head. This is summarized in Figure 6.

If we use the upper back IMU with both *gyr_thresh* and *angle_thresh* to detect onset, the optimal subject-independent thresholds are *gyr_thresh* = 58°/s and *angle_thresh* = 23°. In the within-subject case, the optimal *gyr_thresh* varies (among subjects) from 25°/s to 80°/s while optimal *angle_thresh* varies from 22 to 30°. Similar values (subject-independent optimum: *gyr_thresh* = 56°/s and *angle_thresh* = 23°) were obtained for the lower back IMU. The intersubject variation in optimal *gyr_thresh* is likely due to subjects turning at different speeds.

Furthermore, if we use the upper back IMU with both *gyr_thresh* and *angle_thresh* to detect onset, the majority of detection errors in within-subject crossvalidation are due to 22-degree turns: out of all premature/late detections and false negatives, 72% occur with 22-degree turns, 20% with 45-degree turns, and only 8% with 90-degree turns. Interestingly, this is not the case in subject-independent crossvalidation: there, out of all premature/late detections and false negatives, 31% occur with 22-degree turns, 39% with 45-degree turns and 30% with 90-degree turns.

For the combination of both thresholds (orientation and angular velocity), we additionally evaluated the onset detection for the special case where only larger turns are considered (Table 4). It can be seen that, if only large turns are possible, detection improves for all IMU positions.

### 3.2. Unimpaired Subjects: Turn Direction and Amplitude

**Figure 7.**
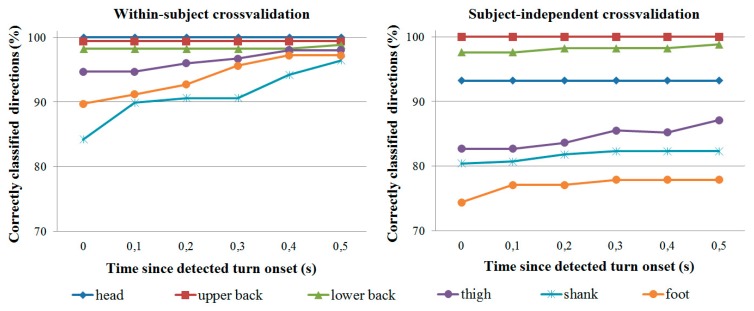
Classification accuracy for turn direction (left/right) classification as a function of time since detected onset. Each line represents a specific IMU.

**Figure 8.**
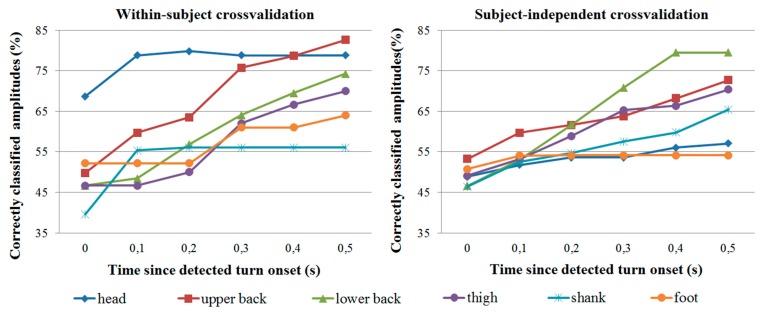
Classification accuracy for turn amplitude (22, 45 or 90 degrees) classification as a function of time since detected onset. Each line represents a specific IMU.

As mentioned, turn direction and amplitude classification was evaluated only for trials where turn onset was correctly detected by the IMU. Classification accuracy is shown in Figure 7 for direction classification and Figure 8 for amplitude classification. In all cases, all three turn amplitudes (22, 45 and 90 degrees) are possible.

In general, the head IMU provides the most reliable direction and amplitude information in within-subject crossvalidation, followed by the two IMUs on the back. In subject-independent crossvalidation, the IMUs on the back are the most accurate. Though not reported in detail, the majority of misclassifications occur with 22- and 45-degree turns. For example, with the upper back IMU, 90-degree turns represent less than 20% of all misclassifications at any given time for both crossvalidation types. At 0.3 s since detected turn onset (or later), 90-degree turns represent less than 10% of all misclassifications for both crossvalidation types.

Care should be taken when comparing turn direction and amplitude classification results from the different IMUs. Since classification is performed only for trials where the turn onset is detected correctly, it is not possible to directly compare e.g., upper back results (where over 85% of turn onsets are correctly detected) with foot results (where less than 75% of onsets are correctly detected). It is therefore also difficult to compare results between different onset detection criteria (e.g., orientation threshold only *vs.* both thresholds), and we have only reported direction and amplitude classification results for the best case when both onset thresholds are used.

### 3.3. Amputee Pilot Trials

**Figure 9.**
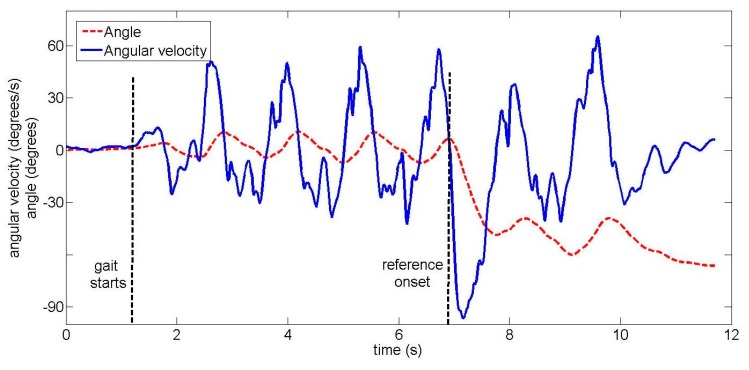
The amputee subject performing a 45-degree left turn, with two signals from the lower back IMU: the angular velocity and the angle around the vertical axis.

**Figure 10.**
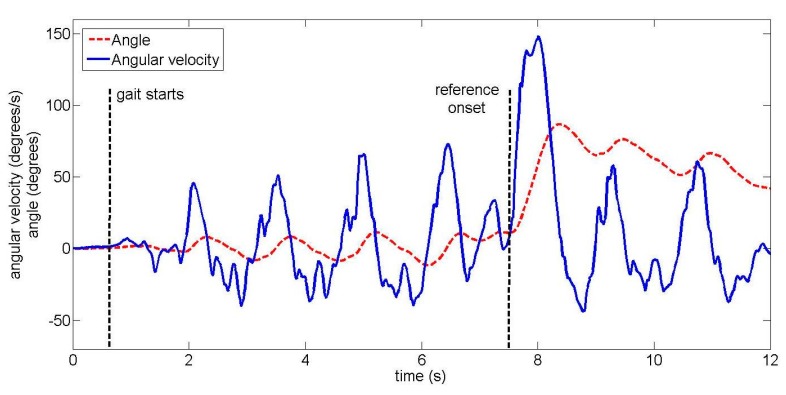
The amputee subject performing a 90-degree right turn, with two signals from the lower back IMU: the angular velocity and the angle around the vertical axis.

**Figure 11.**
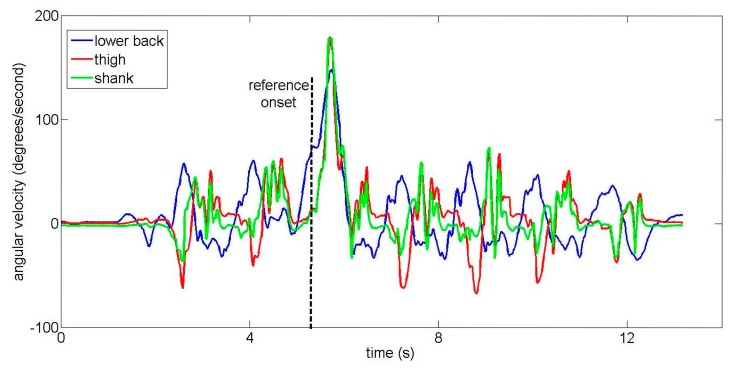
A comparison of angular velocities from the lower back, thigh and shank IMUs when the amputee subject performs a 90-degree right turn.

Two examples of lower back IMU signals from the amputee subject are shown in Figures 9 and 10 for different turns. The amputee turns more slowly than unimpaired subjects, resulting in a smaller angular velocity peak (compare Figure 10 to the equivalent Figure 5 from an unimpaired subject). When the different IMUs were visually compared, turn onset was (subjectively) first and most clearly visible in signals from the lower back IMU for the majority of trials. However, in two trials, turn onset was visible essentially simultaneously for all IMUs. Both of these were 90-degree turns where the amputee slowed down significantly to perform the turn. One such example is shown in Figure 11.

Onset detection was performed using algorithms trained on data from healthy subjects. Results are shown in Table 5 for onset detection using both orientation and angular velocity thresholds. Note that false positives are not possible since there are no trials without a turn, and the other results are given as absolute number of trials rather than a percentage due to the low number of total trials.

Finally, turn direction and amplitude classification were also performed using algorithms trained on data from healthy subjects. Turn direction classification accuracy was similar to accuracy achieved with healthy subjects: at the time of detected turn onset, direction was correctly classified in 8 of 9 trials from the lower back IMU and 6 of 7 trials from the thigh IMU. (As with unimpaired subjects, direction classification was only performed for trials where onset was detected correctly.) However, turn amplitude classification was largely unsuccessful: at the time of detected turn onset, amplitude was correctly classified in 4 of 9 trials using the lower back IMU and 3 of 7 trials using the thigh IMU. 0.5 s after detected turn onset, amplitude was correctly classified in 5 of 9 trials using the lower back IMU and 4 of 7 trials using the thigh IMU.

## 4. Discussion

### 4.1. Optimal IMU Placement

Results from unimpaired subjects indicate that, whatever the onset detection criterion, the best results can be obtained using an IMU placed on the upper or lower back. While there are only small and inconsistent differences between the two IMUs on the back, the IMUs on the legs consistently produce worse onset detection results as well as lower percentages of correctly classified turn directions and amplitudes. The cause for this is not clear, but we believe that it is due to two reasons. The first reason is the top-down turning strategy, where the upper body generally turns earlier than the lower body [21,22]. The second reason is that the trunk begins turning immediately regardless of the leg used to make the turn while the two legs do not turn simultaneously – one lags after the other.

The head IMU is a special case. The majority of subjects look at the endpoint for the current trial even before starting to walk, and vary between looking straight ahead or looking at the endpoint. Thus, while information about the walking direction is visible in the signals from the head IMU, it cannot be used to predict when the turn itself will occur.

We can conclude that, if we wish to detect turn onset rapidly with a single IMU, it should be placed on the trunk rather than the legs. This importance of the upper body during gait is not new and has been previously emphasized in the context of lower limb exoskeletons [13,23], but remains underexplored in gait event detection.

### 4.2. Onset Detection Criteria

For unimpaired subjects, the orientation threshold can be used to correctly detect about 75% of turns using the upper and lower back IMUs in within-subject crossvalidation. The angular velocity threshold correctly detects slightly over 80% of turns using the upper and lower back IMUs in within-subject crossvalidation, but also produces more false positives. Combining both thresholds for onset detection produces the best results, with the highest percentage of correct detections and a low percentage of false positives (Figure 6). Depending on the application requirements, the optimization function presented in Section 2.4 can also be tuned to, for example, ensure no false positives at the cost of missing some small turns.

The amputee, who turns more slowly, exhibits lower angular velocity peaks compared to healthy subjects. This suggests that the angular velocity threshold may be less useful in both amputees and in elderly unimpaired subjects who would also turn more slowly than young subjects. However, the combination of orientation and angular velocity thresholds on the lower back was still able to achieve a reasonable percentage of correctly detected onsets.

It should be noted that, although combining the two thresholds allows turn onset to be detected accurately with respect to an optical reference, this is only important if we are interested in real-time detection where smaller detection delays give an assistive device more time to react. For annotation of offline data, an orientation threshold is sufficient, as has been shown in previous work [18,19]. Neither option is particularly demanding from a computational viewpoint. Angular velocity is obtained directly from the gyroscope while the orientation is calculated via a Kalman filter that requires approximately 120 μs for each processing step on a 1-GHz personal computer, a negligible delay compared to, for example, transmission delays [11].

### 4.3. Subject-Specific or Subject-Independent Training?

The best results were obtained in within-subject crossvalidation, where the algorithms are trained individually for each subject. This implies that, for any online application, the algorithms should also first be trained individually for each subject. Depending on how robust we wish the trained algorithms to be, this training process may be time-consuming as trials of different speeds and turn amplitudes are collected in different conditions. Our own measurement protocol required over 30 minutes for each subject and was conducted at a single walking speed.

The main foreseen application of the algorithm is in assistive devices such as powered prostheses. Such a device would not be used by many people, but even training for one person would be time-consuming. The best approach may be a combination of subject-independent and subject-specific training. For example, the hand prosthesis control approach of Tommasi *et al.* [29] starts with a database of training trials from other subjects. Once it has recorded some signals from the new user, the algorithm identifies one or several similar subjects in the training data and uses them as a starting point for gradual adaptation to the new user over time. A similar algorithm could be used for leg prostheses, shortening the initial training time of the turn detection algorithms.

### 4.4. Choice of Cost Function

The cost function used to train the onset detection algorithm (Section 2.4.1) represents a weakness of the study, as it is somewhat arbitrary. An intuitive alternative would have been to replace it with a more complex way of finding a threshold. We used more complex methods for gait event detection in our previous studies [5,11] but were not satisfied with them for detection of aperiodic events such as gait initiation and termination [11]. We therefore manually created a cost function that would fulfil our goals, but acknowledge that other thresholding methods may also be viable.

The manual approach for onset detection used by the human experts may also bias the algorithm’s results. For example, since the Optotrak markers are placed on the head and trunk rather than the legs, the expert-defined reference onset is more likely to match the lower and upper back IMUs since they are also placed on the legs. We believe that the delays in turn onset between the trunk and legs are much smaller than the 500-ms interval for correct detection, and that this issue therefore did not have a major effect, but it should nonetheless be mentioned.

Within the cost function, some parameters have been chosen ad-hoc based on our own experiences and previous studies. For example, the 500-ms criterion for error computation was chosen based on two things. First, previous studies in arm prosthetics have shown that delays over 300 ms are unacceptable for real-time control [30]. This limit may be even lower for lower limb prostheses where improper control may cause the wearer to fall, though the issue has not been extensively investigated. Second, the two human experts exhibit some uncertainty in defining turn onset, with their estimates varying by more than 200 ms in approximately 5% of trials. The 500-ms value therefore represents a sum of the maximum acceptable delay and an uncertainty in the reference onset timing. However, it is not necessarily an optimal choice, and different values would lead to different performance of the algorithm. Studies with an actual assistive device would be required to estimate the optimal cost function—what kind of delays are acceptable and, for example, whether false negatives or positives should be more strongly penalized.

### 4.5. Generalizability to Impaired and Elderly Users

The main weakness of the presented approach is the limited evaluation, which was done mostly with relatively young, healthy subjects and only a single amputee. Preliminary results from the amputee suggest that turning behavior is not entirely dissimilar in amputees compared to healthy subjects, and onset detection rules can be transferred from healthy subjects to amputees to some degree. However, the amputee does turn more slowly, with lower angular velocity peaks. This suggests that an angular-velocity-based threshold for onset detection may be less useful for amputees and elderly subjects. The slower turning speed may also be the reason why turn amplitude classification was not successful in the amputee.

Furthermore, in a few amputee trials, the turn onset is visible essentially simultaneously for all IMUs (see Figure 11). These trials were 90-degree turns where the amputee had to slow down and nearly stop to complete the turn. As humans utilize different turning strategies during on-the-spot turns [31], this suggests that the relative superiority of IMUs on the back may not generalize to any users whose impairment prevents them from turning without significantly slowing down.

In general, elderly subjects are known to exhibit different turning behavior [21,22]. Additionally, the turning strategy depends on the presence or absence of visual stimuli [31]. Therefore, our results may be limited to only some turn types, and future work will focus on improving robustness for more challenging users and situations.

### 4.6. Use with an Assistive Device

Though our algorithm has not been tested with an assistive device, we can briefly describe the expected application. While the wearer walks with the assistive device (powered prosthesis or orthosis), the algorithm constantly checks whether a turn onset is occurring. Once the onset is detected, the algorithm should immediately notify the device that a turn is occurring, causing it to switch from assisting forward gait to ensuring stability during the turn, similarly to a stumble detection algorithm [14]. The device should also immediately know the turn direction (left/right), as this would greatly affect the assistance provided. Information about turn amplitude, while less critical, may allow the device to modulate the amount of support; for example, by providing less support in the case of smaller turns. However, this assistive behavior needs to be tested directly with an impaired user and assistive device in order to study the interplay between the user and the device and determine the optimal settings.

## 5. Conclusions

This paper builds on previous turn detection algorithms by presenting an optimal setup for real-time turn onset detection during gait using IMUs. Based on our measurements, two conclusions should be drawn. First, IMUs should be placed on the trunk (upper or lower back) rather than the legs for most accurate turn onset detection. Second, a combination of thresholds on orientation and angular velocity provides better results than a single threshold (orientation or angular velocity). Once turn onset has been detected, turn direction can also be easily detected from the IMUs on the back, though amplitude is more difficult to detect. Subject-specific detection thresholds provide better results, though they may be time-consuming to train.

The turn detection algorithm is foreseen for use with assistive devices that need to react to a turn as quickly as possible. Using our cost function, the detection thresholds can be adapted so as to, for example, minimize false positives or false negatives. However, our preliminary test with the amputee highlighted important differences between unimpaired and impaired subjects, such as the slower turning speed. Therefore, further evaluations with target populations and with more difficult situations (e.g., unplanned turns) are required to determine the extent to which our findings can be generalized.

## Figures and Tables

**Table 1.** Turn onset detection using an orientation threshold and various IMU positions in within-subject and subject-independent crossvalidation. CD = correct detection, PLD = premature/late detection, FN = false negatives, FP = false positives, DIFF = mean difference between detected and reference onset.

		**CD (%)**	**PLD (%)**	**FN (%)**	**FP (%)**	**DIFF (ms)**
within-subject	head	**57.7**	33.7	8.6	17.7	−70
	upper back	**76.2**	21.7	2.1	4.8	295
	lower back	**73.8**	24.3	1.8	12.9	233
	thigh	**66.0**	32.6	1.3	14.1	235
	shank	**57.2**	42.5	0.2	17.3	238
	foot	**46.7**	51.2	2.0	25.2	−153

subject-independent	head	**58.1**	31.3	10.7	8.3	179
	upper back	**74.3**	24.9	0.8	11.0	156
	lower back	**68.7**	30.4	0.8	8.1	168
	thigh	**54.5**	44.6	0.0	9.7	213
	shank	**47.1**	52.4	0.0	14.5	199
	foot	**36.7**	56.8	6.5	35.7	−118

**Table 2.** Turn onset detection using an angular velocity threshold and various IMU positions in within-subject and subject-independent crossvalidation. CD = correct detection, PLD = premature/late detection, FN = false negatives, FP = false positives, DIFF = mean difference between detected and reference onset.

		**CD (%)**	**PLD (%)**	**FN (%)**	**FP (%)**	**DIFF (ms)**
within-subject	head	**59.8**	20.0	20.2	14.6	−170
	upper back	**82.4**	8.6	9.1	17.7	21
	lower back	**81.8**	13.9	4.3	11.3	32
	thigh	**63.2**	25.1	11.7	32.3	−10
	shank	**51.3**	23.5	25.0	35.5	−27
	foot	**35.1**	46.4	18.5	40.5	−470

subject-independent	head	**63.2**	14.8	22.0	12.5	−104
	upper back	**73.9**	11.0	15.1	8.1	85
	lower back	**74.3**	14.4	11.2	9.6	63
	thigh	**53.5**	30.0	16.6	38.7	71
	shank	**43.0**	29.1	27.9	32.3	215
	foot	**34.4**	39.4	26.2	45.2	−399

**Table 3.** Turn onset detection using both orientation and angular velocity thresholds, with various IMU positions in within-subject and subject-independent crossvalidation. CD = correct detection, PLD = premature/late detection, FN = false negatives, FP = false positive, DIFF = mean difference between detected and reference onset.

		**CD (%)**	**PLD (%)**	**FN (%)**	**FP (%)**	**DIFF (ms)**
within-subject	head	**63.6**	27.8	0.8	7.7	−204
	upper back	**87.4**	10.4	2.2	4.8	69
	lower back	**83.4**	15.5	1.1	8.1	−5
	thigh	**73.5**	25.1	1.3	10.1	80
	shank	**68.9**	30.7	0.2	9.4	−40
	foot	**66.5**	29.0	4.4	25.7	74

subject-independent	head	**63.6**	33.3	3.1	12.5	−210
	upper back	**74.5**	22.3	3.2	12.9	108
	lower back	**77.3**	21.9	0.8	8.1	56
	thigh	**61.3**	37.5	1.2	6.3	25
	shank	**54.8**	44.7	0.4	7.1	−42
	foot	**45.6**	50.0	4.4	21.4	−78

**Table 4.** Turn onset detection if only larger turns are possible, using both orientation and angular velocity thresholds. CD = correct detection, PLD = premature/late detection, FN = false negatives, FP = false positives, DIFF = mean difference between detected and reference onset.

		**45 and 90 Degrees Only**					**90 Degrees Only**				

		**CD (%)**	**PLD (%)**	**FN (%)**	**FP (%)**	**DIFF (ms)**	**CD (%)**	**PLD (%)**	**FN (%)**	**FP (%)**	**DIFF (ms)**
within-subject	head	**76.2**	21.8	2.1	8.3	−217	**91.2**	8.8	0.0	8.1	−191
	upper back	**93.2**	6.8	0.0	1.6	66	**97.6**	2.4	0.0	0.5	58
	lower back	**89.2**	9.6	1.2	1.6	−39	**97.6**	2.4	0.0	0.5	−6
	thigh	**82.3**	17.3	0.4	5.6	87	**89.6**	10.4	0.0	3.2	77
	shank	**76.3**	23.7	0.0	9.6	−108	**82.4**	17.6	0.0	4.8	−43
	foot	**72.7**	27.3	0.0	19.0	114	**84.3**	15.7	0.0	7.1	42
subject-independent	head	**72.0**	26.4	1.6	8.3	−191	**89.7**	10.3	0.0	8.3	−233
	upper back	**79.5**	20.5	0.0	3.2	85	**93.6**	6.4	0.0	0.0	53
	lower back	**85.2**	14.5	0.4	3.3	−57	**92.8**	7.2	0.0	0.0	15
	thigh	**74.7**	24.9	0.4	9.4	36	**82.4**	17.6	0.0	4.8	89
	shank	**59.4**	40.6	0.0	6.5	−61	**72.0**	28.0	0.0	3.2	−49
	foot	**56.3**	43.6	0.0	18.1	−41	**60.2**	39.8	0.0	9.5	88

**Table 5.** Turn onset detection in the amputee subject using both orientation and angular velocity thresholds, with various IMU positions. CD = correct detection, PLD = premature/ late detection, FN = false negatives, DIFF = mean difference between detected and reference onset.

	**CD (trials)**	**PLD (trials)**	**FN (trials)**	**DIFF (ms)**
lower back	**9**	1	2	143
thigh	**7**	2	3	173
shank	**5**	3	4	199
foot	**3**	5	4	185
